# Dose optimization in newborn abdominal radiography: Assessing the added value of additional filtration on radiation dose and image quality using an anthropomorphic phantom

**DOI:** 10.1016/j.redii.2024.100045

**Published:** 2024-04-24

**Authors:** Annie-Lyne Petit, Rabih Alwan, Julien Behr, Paul Calame, Marion Lenoir, Hubert Ducou le Pointe, Éric Delabrousse

**Affiliations:** a Department of Radiology, hôpital Jean-Minjoz, Besançon, France; b Department of Radiology, hôpital Armand-Trousseau, Paris, France

**Keywords:** Dose reduction, Image quality, Abdominal radiography, Newborn, Phantom, Additional filtration

## Abstract

**Background:**

Abdominal radiographs remain useful in newborns. Given the high radiation sensitivity of this population, it is necessary to optimize acquisition techniques to minimize radiation exposure.

**Objective:**

Evaluate the effects of three additional filtrations on radiation dose and image quality in abdominal X-rays of newborns using an anthropomorphic phantom.

**Material and method:**

Abdominal radiographs of an anthropomorphic newborn phantom were performed using acquisition parameters ranging from 55 to 70 kV and from 0.4 to 2.5 mAs, without and with three different additional filtrations: 0.1 mm copper (Cu) + 1 mm aluminum (Al), 0.2 mm copper + 1 mm aluminum, and 2 mm aluminum. For each X-ray the dose area product (DAP) was measured, the signal-to-noise ratio (SNR) was calculated, and image quality (IQ) was evaluated by two blinded radiologists using the absolute visual grading analysis (VGA) method.

**Results:**

Adding an additional filtration resulted in a significant reduction in DAP, with a decrease of 42% using 2 mm Al filtration, 65% with 0.1 mm Cu + 1 mm Al filtration, and 78% with 0.2 mm Cu + 1 mm Al filtration (*p* < 0.01). The addition of 2 mm aluminum filtration does not significantly decrease the SNR (*p* = 0.31), CNR (*p* = 0.52) or the IQ (*p* = 0.12 and 0.401 for reader 1 and 2, respectively). However, adding copper-containing filtration leads to a significant decrease in, SNR, CNR and IQ.

**Conclusion:**

Adding a 2 mm Al additional filtration for abdominal radiographs in newborns can significantly reduce the radiation dose without causing a significant decrease in image quality.

## 1 Introduction

Although the use of abdominal X-rays is decreasing in adults [1], they continue to play a crucial role in pediatric imaging, particularly in cases of acute abdomen in newborns [2]. In this population, abdominal X-rays are essential for diagnosing and monitoring intestinal obstruction or necrotizing enterocolitis [3], [4], [5] as they provide evidence of bowel loop distension, pneumatosis intestinalis, or pneumoperitoneum. Despite conventional radiography being known for inducing low levels of radiation, the need for repeated examinations in these situations can lead to a cumulative radiation dose and its impact on human health is still subject to debate [6], [7], [8].

Moreover, children, especially newborns, appear to have higher radiation sensitivity and are more susceptible to stochastic radiation effects due to their developing bodies and longer life expectancy postexposure [9], [10], [11], [12], [13]. Therefore, optimizing radiation doses and image quality in newborns remains crucial to obtain X-rays with adequate image quality while keeping the radiation dose as low as reasonably achievable (ALARA) yet diagnostically acceptable (ALADA).

One available solution to reduce the radiation dose is the addition of an extra filtration that removes low-energy photons from the beam, as they contribute to the radiation dose without improving global image quality [14,15]. Copper and aluminum are commonly used materials for this purpose in medical imaging. Numerous studies have demonstrated the benefits of additional filtration for thoracic [16], [17], [18], abdominal X-rays [19,20] and even thoracic CT [21] in adults, as well as for pelvic X-rays in children [22], [23], [24]. In newborns, it has been shown that additional filtration can reduce radiation dose without compromising the quality of thoracic [25], [26], [27], [28] and pelvic X-rays [22]. However, to our knowledge, no study has explored the value of additional filtration for abdominal radiographs in newborns.

Therefore, the aim of this study was to investigate whether the use of additional filtration could reduce the radiation dose of abdominal radiographs in newborns without compromising image quality.

## 2 Materials and methods

### 2.1 Phantom

To conduct this study, X-rays were performed using an anthropomorphic newborn phantom, the Kyoto PH-50B Newborn Whole-Body Phantom PBU-80. This phantom accurately simulates the anatomical features of a newborn abdomen, including the spine and pelvic bones, colon, soft tissues, lungs, and mediastinum, which are typically visible on an abdominal X-ray.

### 2.2 Imaging equipment and acquisition technique

Abdominal radiographs were performed using a Philips DigitalDiagnost X-ray unit, which combines an SRO 33100 ROT 380 X-ray tube with an inherent filtration of 2.5 mm Al/75 Kv and a focal spot (small / large) of 0.6 / 1.2 mm and a maximum voltage of 150 kV. A Trixell DR 35×43 flat panel detector was used. The images were acquired with the anthropomorphic phantom in the antero-posterior lying position, without employing the air gap technique. A source-to-detector distance of 110 cm was used, and the collimation field size at the image receptor was 16 × 15 cm. In accordance with recommendations for pediatric imaging [15,29], no grid was used. All equipment underwent regular quality control tests, and the results were within the specifications provided by the manufacturer.

**Fig. 1:**
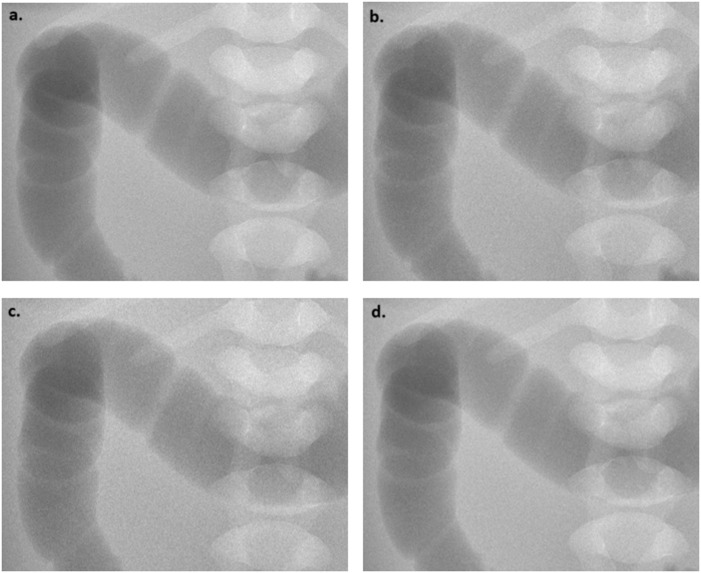
Dose optimization for abdominal radiographs in newborns: example of abdominal X-rays of the phantom all acquired using 60 kV and 2 mAs, with none (a), and with 0.1 mm Cu + 1 mm Al (b), 0.2 mm Cu + 1 mm Al (c) and 2 mm Al (d) additional filtrations, with the use of the same widowing.

A total of 120 abdominal radiographs were obtained with various additional filtrations available on this device: none, 0.1 mm copper + 1 mm aluminum, 0.2 mm copper + 1 mm aluminum, and 2 mm aluminum (Fig. 1). For each group, 40 radiographs were captured, employing five different kilovoltages (55, 57, 60, 66 and 70 kVp), and for each kilovoltage, eight different tube currents (0.4, 0.63, 0.8, 1, 1.25, 1.6, 2 and 2.5 mAs) were applied. These parameter combinations reflect a wide range of settings commonly used in current practice [30].

### 2.3 Radiation dose assessment

The dose area product (DAP) was directly measured by the ionization chamber at the surface of the X-ray tube collimator.

### 2.4 Signal-to-noise ratio (SNR) and contrast-to-noise ratio (CNR)

To calculate the signal-to-noise ratio (SNR) and the contrast-to-noise ratio (CNR), we measured the mean pixel value (PV) in eight circular regions of interest (ROIs) placed on different structures in the phantom's abdomen on a dedicated PACS workstation (Carestream Health, Rochester, NY). Six ROIs were distributed along the colon, one on the right iliac bone and one in the left costophrenic recess, to obtain a representative analysis of images while still focusing on the colon, a major organ in the study of abdominal sonography in newborns. The surface area of each ROI was 100 mm². Additionally, a background ROI was drawn within the abdominal soft tissue where there was no overlap of anatomical structures. The ROIs were consistently positioned across all images. The SNR and the CNR were computed using the following equations:

$$\text{SNR}=\frac{PV_{i}}{SD_{b}}\;CNR\;=\frac{PV_{i}-PV_{b}}{\sqrt{\frac{\sigma_{i}^{2}+\sigma_{b}^{2}}{2}}}$$

Here, *PV_i_* and *PV_b_* represents respectively the mean pixel value in one of the eight ROIs and the mean pixel value in the background ROI, $\sigma_{i}^{2}$ is the mean standard deviation of pixel values in one of the eight ROIs, and $\sigma_{b}^{2}$ is the mean standard deviation of pixel values in the background ROI. $SD_{b}\;$corresponds to the standard deviation of the background ROI [20].

The SNR and the CNR of each image were defined as the average of the SNR and CNR values calculated for the eight different ROIs.

### 2.5 Visual image quality evaluation

As the study was conducted using a phantom, the quality criteria proposed by the European guidelines could not be applied in this context [29]. For example, this phantom lacks kidneys and psoas which are used in the previous criteria. Therefore, we established six evaluation criteria corresponding to the structures of our phantom to ensure a representative analysis of the radiographs: sharpness of the colic wall, reproduction of the colic haustra, sharpness of the bony contour of the vertebrae and iliac bones, reproduction of the costophrenic recess, and overall image quality.

The subjective assessment of image quality (IQ) was performed by two independent radiologists with 3 and 13 years of experience in pediatric imaging. They were blinded to the acquisition parameters and used a visual grading analysis (VGA) method. All abdominal radiographs were reviewed on a dedicated PACS workstation (Carestream Health, Rochester, NY). The radiologists were permitted to adjust the window width and window level as needed. For each X-ray, the VGA process consist of the two radiologists subjectively assigning a quality score ranging from 0 (indicating poor image quality) to 5 (indicating excellent image quality) for each of the six previously mentioned criteria. The radiologists were permitted to adjust the window width and window level as needed. The VGA score of each image were defined as the average of the scores given for each of the six quality criteria.

### 2.6 Statistical analysis

Continuous variables are expressed as mean ± standard deviation (SD). Comparisons between DAP, SNR, CNR and VGA between the four groups were performed using ANOVA and Student's t tests when the distribution was normal and using Kruskall and Wallis and Mann and Withney tests when the data were not normally distributed. The Shapiro-Wilk test was used to examine data normality.

The inter-rater reliability of the VGA was investigated using the intraclass correlation (ICC) with ICC values < 0.50 indicating poor, between 0.50 and 0.75 indicating moderate, between 0.75 – 0.9 indicating good, and > 0.9 indicating excellent reliability [31].

Pearson's r correlation was generated to assess the correlation between visual grading analysis of IQ, SNR and CNR.

An overall P value of less than 0.05 was considered to indicate a statistically significant difference. Statistical analyses were performed using IBM SPSS Statistics, Version 26.0 (IBM Corp., Armonk, NY, USA) and StatiS, Version 19.3.

## 3 Results

### 3.1 Radiation dose

**Fig. 2:**
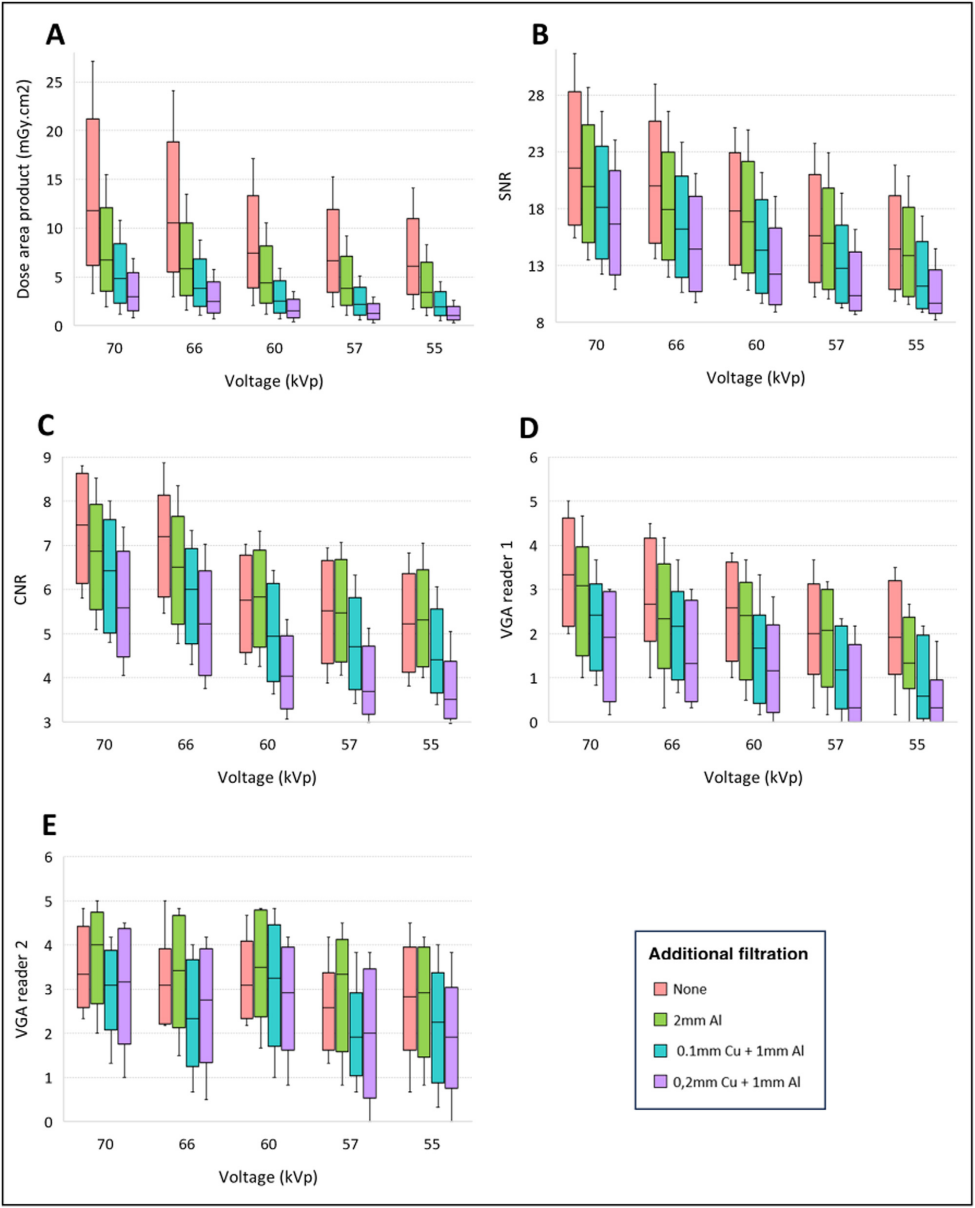
Dose optimization for abdominal radiographs in newborns: variation in dose area product (A), signal-to-noise ratio (B), contrast-to-noise ratio (C) and image quality visual grading analysis for reader 1 (D) and reader 2 (E) according to additional filtration and tube voltage.

The mean DAP for all X-ray images taken without additional filtration was 9.63 ± 6.41 mGy.cm². The use of any additional filtration resulted in a significant reduction in DAP (*P* < 0.001) (Table 1). The addition of 2 mm aluminum led to a 42% decrease in DAP (5.58 ± 3.68 mGy.cm², *P* < 0.001). The reduction was even greater with copper-containing filtration, with a 65% decrease observed with the addition of 0.1 mm copper + 1 mm aluminum (3.61 ± 2.48 mGy.cm², *P* < 0.001), and a 78% decrease with 0.2 mm copper + 1 mm aluminum (2.12 ± 1.61 mGy.cm², *P* < 0.001) (Fig. 2A).

**Table 1:** Dose optimization for abdominal radiographs in newborns: variation in radiation dose and image quality (SNR, CNR and VGA) withe none and different additional filtrations.

Additional filtration	DAP (mGy.cm²)	SNR	CNR	VGA	
				Reader 1	Reader 2
None	9.63 ± 6.41	18.46 ± 5.62	6.20 ± 1.41	2.55 ± 1.23	3.05 ± 1.04
2 mm Al	5.58 ± 3.68 *	17.22 ± 5.12	6.00 ± 1.25	2.13 ± 1.21	3.26 ± 1.24
0.1 mm Cu + 1 mm Al	3.61 ± 2.48 *	14.81 ± 4.67 *	5.33 ± 1.26 *	1.60 ± 1.1 *	2.47 ± 1.21 *
0.2 mm Cu + 1 mm Al	2.12 ± 1.61 *	13.35 ± 4.18 *	4.54 ± 1.22 *	1.15 ± 1.05 *	2.45 ± 1.33 *

DAP: dose area product; CNR: contrast-to-noise ratio; SNR: signal-to-noise ratio; VGA: visual grading analysis. Quantitative data are expressed as mean ± standard deviation.

⁎ indicates a significant *p* value.

### 3.2 Signal-to-noise ratio (SNR) and contrast-to-noise ratio (CNR)

The SNR was not significantly different between the X-rays taken without additional filtration and those taken with 2 mm aluminum, with calculated values of 18.46 ± 5.62 and 17.22 ± 5.12, respectively (*P* = 0.31). However, the SNR was significantly lower when using copper-containing filtration. Specifically, there was a 19.7% decrease in SNR with the addition of 0.1 mm Cu + 1 mm Al (14.81 ± 4.67, *P* = 0.005), and a 27.6% decrease with the addition of 0.2 mm Cu + 1 mm Al (13.35 ± 4.18, *P* < 0.001) (Fig. 2B).

The CNR showed no significant difference between X-rays taken without additional filtration and those taken with 2 mm aluminum, with calculated values of 6.20 ± 1.41 and 6.00 ± 1.25, respectively (*P* = 0.52). However, when copper-containing filtration was used, the CNR significantly decreased. Specifically, there was a 13.9% decrease in CNR with the addition of 0.1 mm Cu + 1 mm Al (5.33 ± 1.26, *P* = 0.01), and a 26.7% decrease with the addition of 0.2 mm Cu + 1 mm Al (4.54 ± 1.22, *P* < 0.001) (Fig. 2C).

### 3.3 Visual image quality evaluation

There was no significant difference in the IQ scores between the X-rays taken without any additional filtration and those taken with 2 mm aluminum for reader 1 (2.55 ± 1.23 vs. 2.13 ± 1.21, *P* = 0.12). For the second reader, the IQ score was even higher with the 2 mm aluminum filtration, although this difference was not significant (3.05 ± 1.04 with no additional filtration vs. 3.26 ± 1.24 with 2 mm aluminum, *P* = 0.401) (Fig. 2D and E).

In contrast, the VGA scores were significantly lower for both readers when using additional filtration containing copper compared to the absence of additional filtration. For reader 1, the VGA score was 1.60 ± 1.1 (a reduction of 37.3%) when adding 0.1 mm copper + 1 mm aluminum (*P* < 0.001) and 1.15 ± 1.05 (a reduction of 54.9%) when adding 0.2 mm copper + 1 mm aluminum (*P* < 0.001). For reader 2, the decrease in IQ was 19% with 0.1 mm copper + 1 mm aluminum (3.05 ± 1.04 vs. 2.47 ± 1.21, *P* = 0.048) and 19.7% with 0.2 mm copper + 1 mm aluminum (3.05 ± 1.04 vs. 2.45 ± 1.33, *P* = 0.029) (Fig. 2D and E).

### 3.4 Interrater agreement

Global interrater agreement was 0.694 (95% CI: −0.072, 0.899; *P* = 0.052). Without additional filtration, the inter-rater agreement was 0.85 (95% CI: 0.211, 0.951; *P* = 0.007). When adding 2 mm aluminum, 0.1 mm copper + 1 mm aluminum, and 0.2 mm copper + 2 mm aluminum filters, the inter-rater agreement was 0.67 (95% CI: −0.039, 0.91; *P* = 0.074), 0.673 (95% CI: −0.078, 0.896; *P* = 0.054), and 0.569 (95% CI: −0.065, 0.862; *P* = 0.092), respectively.

The VGA score and the SNR showed a high positive correlation, with Pearson's r values of 0.97 (95% CI: 0.96, 0.98; *P* < 0.001) for reader 1 and 0.89 (95% CI: 0.85, 0.91; *P* < 0.001) for reader 2. There was also a high positive correlation between the VGA score and CNR with Pearson's r values of 0.96 (95% CI: 0.94, 0.97; *P* < 0.001) for reader 1 and 0.85 (95% CI: 0.80, 0.89; *P* < 0.001) for reader 2. SNR and CNR showed a high positive correlation as well with Pearson's r values of 0.97 (95% CI: 0.96, 0.98; *P* < 0.001)

### 3.5 Comparison of 70 kV with 2 mm aluminum to 57 kV without filtration

The DAP was similar when adding an additional filtration of 2 mm aluminum with a tube voltage of 70 kV compared to the absence of additional filtration with a tube voltage of 57 kV (7.64 ± 4.75 mGy.cm² vs. 7.53 ± 4.69 mGy.cm², respectively, *P* = 0.962).

Conversely, the CNR was significatively higher when adding an additional filtration of 2 mm aluminum with a tube voltage of 70 kV compared to the absence of additional filtration with a tube voltage of 57 kV (6.80 ± 1.24 vs. 5.49 ± 1.14, *P* < 0.05).

The SNR was also higher when adding an additional filtration of 2 mm aluminum with a tube voltage of 70 kV compared to the absence of additional filtration with a tube voltage of 57 kV (20.35 ± 5.42 vs. 16.29 ± 4.91, *P* = 0.14).

VGA was also higher when adding an additional filtration of 2 mm aluminum with a tube voltage of 70 kV compared to the absence of additional filtration with a tube voltage of 57 kV for readers 1 and 2 (2.83 ± 1.3 vs. 2.06 ± 1.16, *P* = 0.231 and 3.75 ± 1.1 vs. 2.58 ± 0.97, *P* = 0.04).

## 4 Discussion

This study is the first to investigate how additional filtration affects radiation dose and IQ in newborn abdominal X-rays. As this population is particularly vulnerable to ionizing radiation, it is important to optimize radiation doses [9], [10], [11], [12], [13]. To achieve this, the French diagnostic reference level (DRL) for newborn abdominal X-rays was set at 20 mGy.cm² [32]. In this study, 98.5% of all X-rays performed were below this level.

This study demonstrates that a reduction in radiation dose for abdominal X-rays is achievable by using additional filtration while maintaining IQ. The role of additional filtration is essentially to reduce skin dose as it removes lower-energy photons from the X-ray beam, which are completely absorbed by the patient and do not contribute to the creation of the radiological image, while increasing the radiation dose [14,15]. This result is consistent with multiple previous studies that have shown a reduction in radiation dose using aluminum, copper, or compound additional filtration for thoracic [16], [17], [18] and abdominal X-rays [19,20] in adults, pelvic X-rays in children [22], [23], [24], and thoracic [25], [26], [27], [28] and pelvic [22] X-rays in newborns.

DAP is a dosimetric indicator used to assess the delivered radiation dose, but it does not correspond to the dose absorbed by the patient. The addition of 2 mm aluminum filtration decreased the DAP by 42% without significantly impacting image quality, as previously demonstrated in thoracic X-rays in newborns [25]. The use of additional filtration containing copper resulted in a greater reduction in DAP, with a decrease of 65% using 0.1 mm copper + 1 mm aluminum and 78% using 0.2 mm copper + 1 mm aluminum. However, the addition of these copper-containing filters led to a significant decrease in image quality. This result can be attributed to the fact that copper filters more photons than aluminum, particularly in the range of photon energies up to approximately 50 kVp for copper compared to approximately 20 kVp for aluminum [15]. practice. The use of copper filtration has been studied in newborn pelvic X-rays [22] and newborn thoracic X-rays [26], [27], [28], and it has been shown to reduce radiation dose without compromising image quality. However, previous studies have shown that additional filtration often requires an increase in tube potential or tube current to maintain image quality. In our study, we kept the acquisition parameters consistent across the four groups defined by the type of additional filtration used. The challenge is to determine if the decrease in image quality affects the detection of signs related to intestinal obstruction and ulceronecrotic enterocolitis, specifically involving intestinal distension, parietal pneumatosis, and pneumoperitoneum. Additional studies must be conducted under conditions that closely reflect clinical routine.

Our results also suggest that adding a 2 mm aluminum filtration and increasing the kilovoltage from 57 to 70 kV could potentially achieve a slightly higher SNR, CNR, and VGA. Indeed, the PDS was similar between a voltage of 57 without filtration and 70 with 2 mm Al and remained below the French diagnostic reference level of 20 mGy.cm2 for newborn abdominal X-rays [32]. This approach could potentially improve image quality while maintaining a low radiation dose, thereby reducing the need for repeated examinations due to inadequate quality. However, further explorations are needed to confirm this hypothesis.

Our study has several limitations. First, we used a newborn phantom for ethical reasons, which allowed for standardization of setup and measurements. However, the resulting image sample may not be representative of abdominal X-rays in the general newborn population. Additionally, the structure of the phantom prevented the use of standardized image quality criteria established by the CEC. Instead, we selected criteria that we deemed relevant for current practice. As a result, the generalizability of our findings to other studies may be limited. Additionally, the phantom does not facilitate the analysis of pathological scenarios as it represents a healthy newborn. Therefore, it is unclear if the observed dose reduction from copper-containing filters significantly affects the detection of signs of intestinal obstruction or necrotizing enterocolitis, which are the primary indications for newborn abdominal X-rays in routine clinical practice. Secondly, the study's statistical power may have been compromised due to the small number of X-rays available for each specific kilovoltage (kV) and milliampere-second (mAs) combination in the four groups with different additional filtration. This limited sample size is particularly relevant in the comparison between 70 kV with 2 mm aluminum filtration and 57 kV without filtration, where some results did not reach statistical significance. Another limitation is the low agreement between raters, indicating that the perception of image quality varies among radiologists. Therefore, we analyzed each reader's data independently. Despite scoring differences, both readers yielded similar results in the statistical tests, suggesting consistency in image ranking.

## 5 Conclusion

The radiation dose of newborn abdominal radiographs could be significantly reduced by adding a 2 mm aluminum additional filtration without impairing image quality. The use of a filtration containing copper resulted in greater dose reductions but did not allow for the maintenance of image quality. Nevertheless, additional studies are necessary to assess whether this reduction in image quality impacts the detection of signs of intestinal obstruction or necrotizing enterocolitis in newborn's abdominal X-rays in routine clinical practice.

## Ethics approval, consent to participate and consent for publication

Not applicable as the study was conducted on an anthropomorphic phantom.

## Funding

There was no funding for this study.

## CRediT authorship contribution statement

**Annie-Lyne Petit:** Data curation, Formal analysis, Investigation, Writing – original draft, Writing – review & editing, Visualization. **Rabih Alwan:** Conceptualization, Methodology, Project administration. **Julien Behr:** Data curation, Investigation. **Paul Calame:** Formal analysis, Validation, Writing – review & editing, Visualization. **Marion Lenoir:** Conceptualization, Supervision. **Hubert Ducou le Pointe:** Supervision. **Éric Delabrousse:** Supervision.

## Declaration of competing interest

The authors declare that they have no competing interests.
